# Trauma Registry Data as a Policy-Making Tool: A Systematic Review on the Research Dimensions

**DOI:** 10.30476/BEAT.2021.91755.1286

**Published:** 2022-04

**Authors:** Mohammadreza Mobinizadeh, Farzan Berenjian, Efat Mohamadi, Farhad Habibi, Alireza Olyaeemanesh, Kazem Zendedel, Mahdi Sharif-Alhoseini

**Affiliations:** 1 *National Institute for Health Research, Tehran University of Medical Sciences, Tehran, Iran*; 2 *Department of Health Economics and Management, School of Public Health, Tehran University of Medical Sciences, Tehran, Iran*; 3 *Health Equity Research Center (HERC), Tehran University of Medical Sciences, Tehran, Iran*; 4 *National Institute for Health Research and Health Equity Research Center (HERC), Tehran University of Medical Sciences, Tehran, Iran*; 5 *Cancer Research Center, Cancer Institute, Tehran University of Medical Sciences, Tehran, Iran*; 6 *Sina Trauma and Surgery Research Center, Tehran University of Medical Sciences, Tehran, Iran*

**Keywords:** Trauma, Registry, Policy-making, Research priority setting

## Abstract

**Objective:**

To review the research dimensions of trauma registry data on health policy making.

**Methods:**

PubMed and EMBASE were searched until July 2020. Keywords were used on the search process included Trauma, Injury, Registry and Research, which were searched by using appropriate search strategies. The included articles had to: 1. be extracted from data related to trauma registries; 2- be written in English; 3- define a time period and a patient population; 4- preferably have more details and policy recommendations; and 5- preferably have a discussion on how to improve diagnosis and treatment. The results obtained from the included studies were qualitatively analyzed using thematic synthesis and comparative tables.

**Results:**

In the primary round of search, 19559 studies were retrieved. According to PRISMA statement and also performing quality appraisal process, 30 studies were included in the final phase of analysis. In the final papers’ synthesis, 14 main research domains were extracted and classified in terms of the policy implication and research priority. The domains with the highest frequency were “The relationship between trauma registry data and hospital care protocols for trauma patients” and “The causes of Disability Adjusted Life Years (DALYs) due to trauma”.

**Conclusion:**

Using trauma registry data as a tool for policy-making could be helpful in several ways, namely increasing the quality of patient care, preventing injuries and decreasing their number, figuring out the details of socioeconomic status effects, and improving the quality of researches in practical ways. Also, follow-up of patients after trauma surgery as one of the positive effects of the trauma registry can be the focus of attention of policy-making bodies.

## Introduction

Injury is a major cause of mortality and disability all over the world. Based on the 2019 Global Burden of Disease Study, the percentage of Disability Adjusted Life Years (DALYs) injuries was 2.9 (2.6-3.2) [1], and there were 4,260,493 injury deaths in 1990, which increased to 4,484,722 deaths in 2017 [2]. Road injuries alone are the 7^th^ leading cause of DALYs globally, ahead of diabetes (10^th^), tuberculosis (12^th^), and malaria (14^th^) [1].

Regarding the burden of trauma disease, the World Health Organization developed a guideline on trauma care quality improvement (QI) [3]. This guideline has emphasized the development of hospital trauma care and assessment of the care quality by implementing quality assessment programs and using specific information systems introduced as one of the best tools for quality assessment. The information systems of different organizations are considered as an important tool in making fundamental policies and decisions. Relying on facts and statistics that are considered raw data, these systems can provide analyzed and helpful information to the management of the organization. The 20^th^ century is one of the keys and critical periods in the evolution of health systems worldwide, during which the first information instruments in health systems were formed. National health systems were established in most countries between 1940 and 1960. The ultimate goal of health information systems is to improve the data and information derived from them in decision-making from the implementation level to policy-making level [4].

A trauma registry is deﬁned as “a disease-speciﬁc collection composed of a ﬁle of uniform data elements that describe the injury event, demographics, pre-hospital information, diagnosis, care, outcomes, and costs of treatment for injured patients”[5]. As a kind of health Information System (HIS), trauma registries have been an important component of trauma systems for decades [4]. They have been essential to improve record-keeping methods and are frequently used to elaborate the beneﬁts of trauma systems [4]. In the meantime, recording trauma cases could have many benefits includes quality assessment, setting a framework for developing and evaluating prevention strategies, and optimizing the policy-making process in this area which lead to a reduction in deaths and casualties due to trauma [6].

Taking advantage of such registries have a tremendous effect on the burden of trauma mortality and morbidity especially in low and middle-income countries (LMICs), where the burden of injury remains a big challenge [3]. There are some barriers to the data implementation and utilization of the trauma registries, namely poor data quality, lack of technology, infrastructure, funding, or human resources, and administrative difﬁculties [5], all of which make these registries inefficient. Researchers have drawn the conclusion that registering trauma cases significantly reduces the mortality of major trauma patients in the hospital and after injuries. However, it will not be possible to use these systems without having a set of injury codes as well as inclusion criteria and variables [6].

Therefore, it is crucial to identify the function of these registries and find out how policymakers can use them for controlling and decreasing the mortality and morbidity of trauma disease with regards to the importance and barriers of the data implementation and utilization in trauma registries. To achieve these objectives, it is crucial to determine the current status and well-document the existing studies, plans, and synthesized knowledge about trauma registries data. Therefore, this study purposes to provide a systematic review on applications of trauma registry data. The results of this study will provide evidence for countries especially LMICs and to realize that research dimensions of the trauma registry data could be more effective in health policy-making.

## Materials and Methods

This systematic review was conducted according to the Preferred Reporting Items for Systematic Review and Meta-analysis statement [7] and a narrative approach for synthesizing the evidence.

Search Strategy

*) Search strategy for PubMed database:

((((trauma) OR (trauma) [MeSH Terms])) OR ((injury) OR (injury [MeSH Terms])))) AND ((registry) OR (registry [MeSH Terms])) AND (Research) 

*) Search strategy for EMBASE database:

(‘trauma’ / exp OR trauma OR ‘injury’ / exp OR injury) AND (‘registry’ / exp OR registry OR record) AND research AND [article] 

Inclusion Criteria 

The included articles had to: 1. be extracted from data related to trauma registries which were established in the hospital setting; 2- be written in English; 3- define a time period and a patient population; 4- preferably have more details and policy recommendations; and 5- preferably have a discussion on how to improve diagnosis and treatment.

Exclusion Criteria 

The studies which was performed in a pre-hospital setting or related to assess a trauma diagnostic test or therapeutic intervention were excluded from the study. 

Quality Assessment

The qualitative critical appraisal and cohort studies was done using critical appraisal skills program (CASP). Hence, quality appraisal of the included studies was done via a self-made form including some concepts of CASP checklists for qualitative and cohort studies. Following items were considered for quality appraisal: “Is the purpose of the study carefully stated?”, “Is the study design in line with the purpose of the study?”, “Have the consequences and results of the study been carefully examined?”, “Are the practical and operational points of the study stated?” Three states for each item were considered: “Yes”, “No” and “Not clear”.

Study Selection

The systematic search was performed on PubMed and EMBASE using keywords related to trauma registry without language or date restriction on July 1, 2020. Also reference lists of relevant reviews were checked. Two reviewers screened the titles and abstracts independently. If there was any disagreement between the two reviewers, the principal investigator was consulted to resolve the disagreement. Duplicate articles, editorials, commentaries, and reviews were excluded.

Data Extraction

The extracted data from the included studies were qualitatively analyzed through thematic synthesis and comparative tables. The main themes in this research were the most frequent “research domains” related to trauma registry systems around the world.

## Results

**Fig 1:**
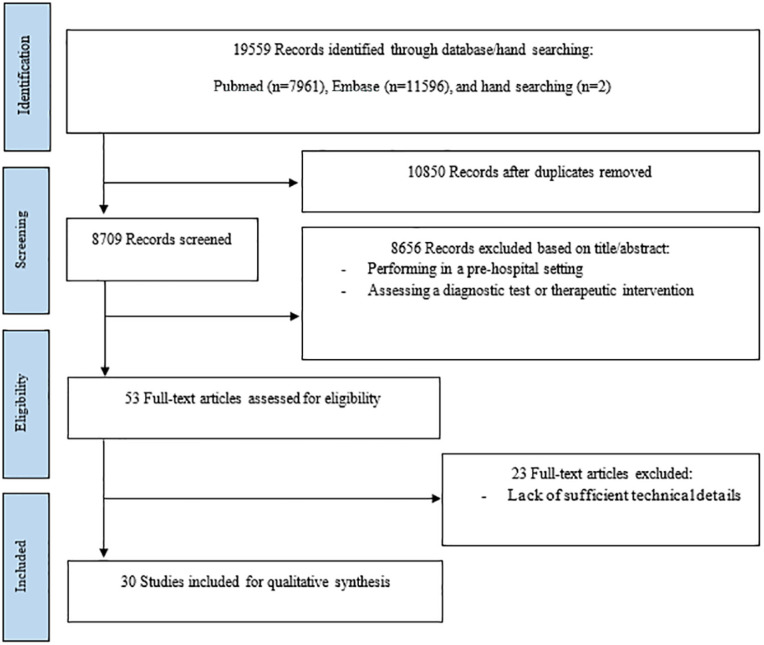
Data Extraction Process (PRISMA)

The initial articles included 7961 from PubMed, 11596 from EMBASE, and 2 articles from hand searching of relevant reviews. After removing irrelevant and duplicate articles, 53 articles remained of which 30 met the inclusion criteria, and entered in the final review (23 papers were excluded in this stage due to lack of sufficient technical details). Figure 1 summarizes the flowchart of our literature review and data extraction process based on Preferred Reporting Items for Systematic Reviews and Meta-Analyses (PRISMA) protocol. 

We synthesized the 30 included studies (Table 1) to identify how trauma registries can be used in policy-making. Based on our synthesis, 14 main research domains were extracted and classified in terms of the policy implication and research priority (Table 2). All included papers had acceptable quality based on the results of quality appraisal form (Table 1). Hence each domain was introduced and described in this manuscript.

**Table 1:** A summary of extracted data from included studies

**No.**	**Basic characteristics of the studies**		**Criteria for quality appraisal of the articles**			
	**Title**	**Publication Date**	**Is the purpose of the study carefully stated?**	**Is the study design in line with the purpose of the study?**	**Have the consequences and results of the study been carefully examined?**	**Are the practical and operational points of the study stated?**
1	Epidemiology of severe trauma [8]	2014	Not Clear	Not Clear	Yes	Yes
2	Bicycle-Related Injuries in Pediatric Patients [9]	2018	Yes	Yes	Yes	Not Clear
3	The Australian Trauma Registry [10]	2018	Not Clear	Yes	Yes	Not Clear
4	Gun trauma and ophthalmic outcomes [11]	2018	Yes	Yes	Yes	Yes
5	Correlation between field triage criteria and the injury severity score of trauma patients in a French inclusive regional trauma system [12]	2019	Yes	Yes	Yes	Not Clear
6	The number of displaced rib fractures is more predictive for complications in chest trauma patients [13]	2017	Not Clear	Yes	Yes	Not Clear
7	The price of personal mobility: burden of injury and mortality from personal mobility devices in Singapore - a nationwide cohort study [14]	2019	Yes	Yes	Yes	Not Clear
8	Is there an association between female gender and outcome in severe trauma? A multi-center analysis in the Netherlands [15]	2019	Yes	Yes	Yes	Yes
9	A prospective stepped wedge cohort evaluation of the new national trauma team activation criteria in Sweden – the TRAUMALERT study [16]	2019	Yes	Yes	Yes	Yes
10	Penetrating Colon Trauma Outcomes in black and white males [17]	2018	Yes	Yes	Yes	Yes
11	Strategies for successful trauma registry implementation in low- and middle-income countries—protocol for a systematic review [18]	2018	Yes	Yes	Yes	Not Clear
12	Injury coding in a national trauma registry: a one-year validation audit in a level 1 trauma center [19]	2019	Yes	Yes	Yes	Not Clear
13	The spectrum and outcome of blunt trauma related enteric hollow visceral injury [20]	2018	Not Clear	Yes	Yes	Yes
14	Evaluation of trauma registry data in Asia region [21]	2001	Yes	Not Clear	Yes	Yes
15	Presenting an evaluation model of the trauma registry software [22]	2018	Yes	Yes	Yes	Yes
16	Trauma registry implementation in low- and middle-income countries: challenges and opportunities [5]	2018	Yes	Yes	Yes	Yes
17	Developing Australia’s first statewide trauma registry: What are the lessons? [23]	2004	Yes	Not Clear	Yes	Yes
18	Pediatric disaster preparedness: The potential role of the trauma registry [24]	2009	Yes	Yes	Not Clear	Yes
19	Trauma registry data validation: Essential for quality trauma care [25]	2006	Yes	Not Clear	Yes	Yes
20	Systematic review of trauma system effectiveness based on registry comparisons [26]	1999	Yes	Not Clear	Yes	Yes
21	Trauma registries: What is the experience in developing countries? [27]	2013	Yes	Yes	Yes	Not Clear
22	Global trauma registry mapping: A scoping review [28]	2012	Yes	Yes	Yes	Yes
23	Trauma Registries: History, Logistics, Limitations, and Contributions to Emergency Medicine Research [29]	2011	Yes	Not Clear	Yes	Not Clear
24	State Trauma Registries as a Resource for Occupational Injury Surveillance and Research: Lessons From Washington State, 1998-2009 [30]	2016	Yes	Yes	Yes	Yes
25	Trauma Surveillance and Registry Development in Mozambique: Results of a 1-Year Study and the First Phase of National Implementation [31]	2019	Yes	Yes	Yes	Yes
26	Trauma registry comparison: six-year results in trauma care in Southern Finland and Germany [32]	2014	Yes	Yes	Yes	Not Clear
27	Developing a low budget trauma registry [33]	2019	Yes	Not Clear	Yes	Not Clear
28	Canadian Benchmarks in Trauma [34]	2007	Yes	Yes	Yes	Yes
29	Exploring data sources for road traffic injury in Cameroon: Collection and completeness of police records, newspaper reports, and a hospital trauma registry [35]	2017	Yes	Yes	Yes	Yes
30	Routine follow up of major trauma patients from trauma registries: What are the outcomes? [36]	2006	Yes	Yes	Yes	Not Clear

**Table 2:** The main research domains extracted from studies and their frequency

**Frequency**	**Domains**	**No.**
8	Determining the relationship between trauma registry data and hospital care protocols	1
5	Determining the causes of DALYs due to trauma	2
3	Carrying out economic evaluations and assessment of effectiveness concerning the existence of a trauma registry system	3
2	Investigation of the cases of trauma in children and adolescents	4
2	How triage checklists for patients with trauma are periodically reviewed?	5
2	Investigation of trauma injuries using existing severity calculation scales	6
2	Estimation of the burden of trauma and determining its risk factors	7
1	Calculation of DALYs due to trauma	8
1	Incidence of trauma (Based on Income Status)	9
1	Investigating the distribution of trauma events based on gender	10
1	Investigating cases of trauma caused by non-motorized vehicles such as bicycles and scooters	11
1	Investigating the distribution of trauma events based on the type of race and ethnicity	12
1	How trauma codes are constantly reviewed?	13
1	Assessing the mortality rate in the provinces participating in the trauma registry network	14

Determining the Relationship between Trauma Registry Data and Hospital Care Protocols 

Australia had the highest number of road deaths. In this country, the Australian National Trauma Registry Consortium has been established since 1993. The development of this project in Australia continued in line with the country’s road safety strategy from 2011 to 2020. Finally, this national system began its official activity in 2016 with the receipt of government funding. The registered dataset was streamlined between 2017 and 2018 with the exact date of each trauma case. The Australian National Trauma System also records additional events such as falls from ladders. These studies have led to the development and implementation of 19 models to improve care delivery quality [10].

Trauma Registry in the United States has been used to change the law, promote trauma prevention, and assess the effectiveness of the trauma system. Australia established its first national trauma registry system in 2001. Trauma records are reviewed at local centers to increase the quality of treatment. In Australia, each province has a file that covers all hospitals in the province [36].

In the United States, the development of trauma registry coincided with the establishment of trauma registry centers in the 1970s, although comprehensive information was not yet available. In 1982, the American College of Surgeons Committee on Trauma (ACS COT) coordinated the first comprehensive study in this respect. The achievements of the trauma registry includes evaluation and improvement of patient care, identification of opportunities for injury prevention initiatives, documentation of the medical, socioeconomic effects of trauma, and research [36].

Trauma data from Finland and Germany between 2006 and 2011 were reviewed and found to be similar in service delivery and service quality in the two countries. This comparison of the two registries was a method for quality control in trauma centers [33].

Trauma registry provides useful epidemiological injury information that is effective to improve the quality of services. In LMICs and due to trauma, the rate of death is high due to two reasons, including violation of traffic laws and lack of a pre-hospital trauma registry system for prevention. Therefore, the first step is to prepare and design a national trauma registry system [33].

In trauma registries, examining outcomes other than mortality can increase the quality of care and standards of hospital management, appropriate health systems, and better resource allocation. Among other things, it is possible to follow up patients after trauma surgery [23].

The burden of trauma in developing countries is far greater than that in other countries. Although low-income countries bear a high burden of trauma, high-income countries do not enjoy high-quality trauma records. In 2004, the World Health Organization published guidelines for essential trauma care, and in 2009 published guidelines to focus on increasing the quality of care for trauma patients [5].

Criteria in the Canadian Trauma Registry are used to evaluate the quality of care. These results can be used to assess patient’s survival using the severity of the injury assessment method. With regular updates, these data can be used to continuously assess trauma outcomes, quality improvement, and trauma care research in Canada [34].

Determining the Causes of DALYs Due to Trauma 

Occupational Injury

Occupational injuries place a heavy burden on workers, employers, and the US Health Care System in general. Sears and Bowman (2016) report that over $ 250 billion is spent annually on occupational injuries, far more than the $ 219 billion costs of cancer. Despite the preventive measures against it, occupational injury still tops the pyramid of American workers’ mortality and disability. In 2011, the National Institute for Occupational Safety and Health in the United States reported 2.9 million American workers in emergency services due to occupational injuries, of which about 150,000 were hospitalized. More than 4,300 American workers lost their lives due to occupational injuries in 2012. Surveillance of these injuries is useful for their prevention, treatment promotion, and effective policy-making. Sometimes there are restrictions on registering resources, such as preventing workers or employers from registering information to prevent certain losses. According to Sears and Bowman (2016), occupational injury surveillance will have a significant impact on the health objectives set for 2020. In their study, Sears and Bowman (2016) focused on the World Health Organization’s injury pyramid, comparing occupational injuries and the available data, and found no data resources to cover all injuries occurring at work [30].

Accident Injuries

The road trauma surveillance system is the mainstay of injury control efforts. In high-income countries, the data source is the police, while in low- and middle-income countries (LMICs), there is no clear and controllable sources for this data. In Cameroon, for example, the trauma registry records the complete information while the police record the most first-hand information in the initial encounter with the accident. Investing in a hospital-based trauma registry may provide the best surveillance over road traffic injuries in some LMICs. However, police reports and newspapers may serve as alternative data sources if specific information is required [35].

Regarding the rate of complications due to rib fractures, the most traumatic injuries are related to motorcycle accidents. Those who suffered a fracture of 3 or more ribs also experienced chest complications later. As a result and as the number of rib fractures increases, the risk of pulmonary complications and death also increases [13].

Injuries Caused by Conflicts

Injury is one of the most common public health problems globally, which kills an average of 5.8 million people annually in the world. It is the main cause of death among people under 45 years, in both men and women, and the loss of personal abilities and disability. Most of the trauma burden is imposed upon LMICs, where it accounts for more than 90% of deaths due to injury. According to studies and guidelines published by the World Health Organization, what distinguishes high-income countries from LMICs in this respect are the proper planning and implementation of careful policies to improve prevention and quality of care in high-income countries than LMICs [23].

In 2014, 33,599 people in the United States died from gunshot wounds, accounting for 16.8% of all traumatic deaths that year. Unfortunately, one of the reasons that the goals of the national trauma system are not realized in this regard, is the lack of sufficient funding to prevent gun damage which is also hindered by legal issues. Therefore, the number of studies on gun trauma is very small, and the studies of eye trauma due to the use of guns are far fewer. Of the 915 patients admitted to hospitals in New York with gunshot wounds, 27 had eye injuries. The information of 22 cases was available, and 4 of them died due to bleeding. This information was classified into different causes by trauma tables [11].

Carrying Out Economic Evaluations and Assessment of Effectiveness Concerning the Existence of a Trauma Registry System 

The cost of recording, maintaining, and using trauma data varies from country to country, so much so that in 2015, the maintaining cost of this information was estimated at 95$ per patient in high-income countries. However, it can be controlled by the allocation of appropriate budgets [19].

In the perspective of the world health system, maintaining the desired quality is one of the basic pillars. Still, in order to calculate costs and allocate resources, recording and using the information output of trauma patients is very important and critical. These cost reports can provide useful information for payers. Recorded information includes demographic information, injury, and severity, and pre-hospital care. Estimates suggest a cost of $ 140-100 per patient record in the range of 500-700 patients per year [23].

Many studies only examine the recorded data and analyze them to prevent accidents and evaluate medical care while serving trauma patients. However, other studies evaluating the effectiveness of the trauma system and the related data in terms of mortality risk reported that this rate was significantly reduced by 15 to 20% [26].

Investigation the Cases of Trauma in Children and Adolescents

Regarding the trauma registry in adolescents younger than eight who were injured during cycling in Singapore, 733 cases of trauma were investigated, of which 81 were children or adolescents with a mean age of 13 years, including 72 boys and 9 girls. Having an accident with other vehicles was the most common cause of trauma (69.1%), whereas loss of balance while cycling was the least common cause (2.6%). Findings like these provide people who are most affected by this type of trauma the greatest benefit in the future prevention of these events in the light of the right policies used in these studies. Interventions like this can be legislative or non-legislative. Legislative injury prevention tools can include the mandatory use of helmets and other safety equipment for children 12 years of age or older. Such rules can increase the use of safety equipment. In local contexts, non-legislative interventions aimed at preventing injury can be bicycle safety education as a mandatory part of the “Physical Education” syllabus in high school [9].

Children suffer from many injuries during an accident, and it may not be possible to transfer them to optimal trauma centers. As a result, they may not receive appropriate care. Therefore, appropriate policies must be made in order to reduce such failures. It is recommended that child care guidelines be well incorporated into the main trauma guidelines throughout a national trauma system [25].

How Triage Checklists are Periodically Reviewed for Patients with Trauma?

The French triage model based on the Vittel criteria algorithm should be evaluated and revised according to the new criteria. This algorithm leads to an over triage of trauma patients in trauma centers, which increases costs and overcrowding in these specialized centers. As a result, the quality of work is reduced, and those who do not need specialized services use these services and resources. For example, patients with an injury severity score (ISS) above 15 must go to a specialized Level I trauma center. Next, the mortality rate of these patients is studied within 30 days in the intensive care unit [12].

Sweden’s trauma criteria examine the casualty during the accident and before referral to the hospital to ensure that the patient’s conditions in the hospital are fully optimized. The ultimate goal of this evaluation is the optimal use of the golden time to save patients’ lives [16].

Investigation of Trauma Injuries Using Existing Severity Calculation Scales

In Singapore, trauma is the leading cause of hospitalization and death. Singapore National Trauma Registry System was established in 2011, and it covers all public hospitals in the country. Its primary purpose is to evaluate the number of injuries with an injury severity score higher than 9. (ISS> 9) [14].

The trauma registry system is the key to improve the quality of health care in this respect. Meanwhile, although developing countries have developed their registry systems, they have faced resource constraints. Most of the articles dealing with this subject are related to Iran, which was used by China, Jamaica, and Uganda. The most widely used scale to determine the severity of the injury was the ISS score [27].

The Burden Estimation of Trauma and Determining Its Risk Factors 

According to the results of a study on the global burden of diseases, 4.7 million people died of trauma in 2015, more than half of whom lived in low-income countries. Hence, the need to establish a national trauma registry system and enhance the quality of assessments performed based on will be essential [24].

Calculation of DALYs Due to Trauma 

In this regard, 29% of the total trauma cases were related to road accidents, 12.6% to falls from heights, and 9.16% to personal violence. Data show that road accidents and different kinds of violence have been the cause of death among 25-35 years old. However, these accidents become relatively less common after the age of 45, and after the age of 75, falls from heights become the main cause of death. In Spain, the National Statistics Institute recorded 40,950 deaths in 2012, 3.5% of which was related to external injuries that caused the death of the individual [8].

Incidence of Trauma (Based on Income Status)

 Evidence suggests that the trauma cases distribution increases in LMICs, reaching 90% of the world’s population [8].

Investigating the Distribution of Trauma Events Based on Gender

Different results have been obtained from numerous studies conducted on gender differences and survival after severe trauma. Some studies have shown that females are protected from bleeding and sepsis after major trauma. However, gender alone did not account for the increase or decrease in the chances of survival. Men having a trauma score equal to or greater than 25 and being 50 or younger were 27% more likely to die than their women counterparts. Women having injury scores equal to or greater than 25 and being 50 or younger were more likely to die in hospital than men. This study concluded that in females, there is a significant protective effect on the production of new cells in the body. Also, minor trauma usually has no acceptable effect in this study, whereas, this significant difference between men and women can be seen in major trauma [15].

The Trauma Cases Investigation Caused by Non-motorized Vehicles Such as Bicycles and Scooters

The level of trauma inflicted on people is lower than motorized personal mobility devices due to non-motorized vehicles such as bicycles, motorized and non-motorized scooters. Most of the victims of non-motorized vehicles were men with an average age of 33 years. The common type of vehicles was motorcycles and electronic scooters. The mean ISS was 2, but about 25% of those with serious injuries with an ISS equal to or greater than 9 had an average hospital stay of 3 days, and only 1 in 6 patients required surgery. Out of the 614 patients studied, six died [14].

Investigating the Distribution of Trauma Events Based on the Type of Race and Ethnicity 

In the case of trauma patients, data from the National Trauma Database do not show a significant difference between blacks and whites in terms of mortality and outcomes. The National Trauma Database surveyed men with penetrating abdominal trauma aged 14 years or older from 2010 to 2014. The primary results of the study were concerned with stoma formation and rehabilitation, but the secondary results dealt with postoperative survival. Of the 7324 studied patients, 4,916 were black, and 2,916 were white. Whites and blacks were almost similar in terms of stoma formation. However, more white patients were covered by insurance compared with their black counterparts, with 37.1% of black patients as opposed to 29.9% of white patients having direct out-of-pocket payments. Also, gunshot wounds were more common in black people (88.3%) than in white people (70.2%) [17].

How Trauma Codes Are Constantly Reviewed?

The trauma registry system codifies injuries to individuals based on their severity and related scales. The severity of the injury is then calculated based on the obtained scores. These examinations include all the information contained in the electronic records and diagnostic images. Injury codes should be reviewed for validity, and then missing codes needed for trauma are examined. The goal is to validate the injury codes. An expert encryption team determines the injury codes and renders them as the input of the trauma registry system. Trauma programmers set pre-determined criteria in the national trauma registry system. As a result, registry codes and criteria calculate the severity of the injury and determine its score [19].

Assessing the Mortality Rate in the Provinces Participating in the Trauma Registry Network 

Trauma is one of the most important causes of death worldwide, with 5.8 million people dying annually and many more becoming disabled. Most of the injuries occur in low and middle income countries. In general, the death rate of trauma patients in countries with a national trauma system is six times less than that in countries without a registry system [22]. 

## Discussion

In 2010, trauma was the leading cause of death among men and women aged 15-49 years, accounting for 6.23% of all deaths. Therefore, the existence of a trauma registry system at a national level is desperately felt [37]. Also, based on the experiences of different countries, the implementation of comprehensive trauma systems on a regional basis has significantly reduced mortality and complications from trauma. These systems have a basic need for continuous monitoring. In this way, in addition to evaluating the quality of response of medical staff, the system can provide appropriate solutions to prevent accidents and provide clinical solutions that will be of interest to policymakers and physicians [6]. We retrieved 30 studies to identify the most common functions of trauma registry data globally and extracted 14 domains from these studies. 

Calculating the burden of trauma and determining its risk factors was one of the functions of trauma registry data. Trauma is a worldwide cause of death or disability that varies along a national-local continuum. Risk factors for trauma are related to the lifestyle of individuals and society. This can have a significant contribution to policy-making and estimating the burden of disease [8].

Registering trauma cases and having an appropriate pattern of data recording related to them can have many positive effects, such as reducing accident-related deaths, preventing injuries before an accident, and improving policy-making in health systems. Examining the patterns related to the injury before sending the patient to medical centers, observing service standards immediately after the accident, paying attention to the criteria related to patient discharge after recovery and returning home, and measuring and comparing the rate of those recovered in different countries can have a great impact on preventing injuries caused by trauma in the country.

In conclusion, recording trauma cases and a trauma registry establishment can have beneficial effects on the trauma systems in different countries. These effects can includes reducing accident-related deaths, preventing injury, or improving health system policy. Future studies are recommended to examine patterns related to the injury before sending the patient to medical centers, standards of service immediately offered after the accident, criteria related to patient discharge after recovery and returning home, the rate of recovery, and quality of services in different countries.

## Limitations 

The most important limitation in this review was related to the selection of quality appraisal checklist, due to the type of included papers which was mainly similar to qualitative and cohort frameworks, researchers were forced to create a self-made checklist for quality appraisal. 

Also, access to the full text of some papers was limited, hence researchers was forced to exclude them due to this reason. 

## Declarations

### Ethics approval and consent to participate:

This research was confirmed by ethics committee of Sina Trauma and Surgery Research Centre, Tehran University of Medical Sciences, Tehran, Iran with The ethics code: 99-01-93-390.

### Consent for publication:

All authors have seen and agree with the contents of the manuscript

### Conflict of interests:

The authors declare that they have no competing interests.

### Funding:

 This research was funded by Sina Trauma and Surgery Research Center, Tehran University of Medical Sciences (Ref. Code: 99-1-93-46973).

### Authors' contributions:

All authors contributed to design, data collection and drafting of this manuscript equally.

### Acknowledgment:

This research was supported by Sina Trauma and Surgery Research Center, Tehran University of Medical Sciences (Ref. Code: 99-1-93-46973). 
